# Expansion of Vertebrate Pest Exclusion Fencing and Its Potential Benefits for Threatened Fauna Recovery in Australia

**DOI:** 10.3390/ani10091550

**Published:** 2020-09-01

**Authors:** Deane Smith, Kristy Waddell, Benjamin L. Allen

**Affiliations:** 1 Institute for Life Sciences and the Environment, University of Southern Queensland, Toowoomba, QLD 4350, Australia; benjamin.allen@usq.edu.au; 2 School of Arts, Social Sciences and Humanities, Swinburne University of Technology, Hawthorn, VIC 3122, Australia; kwaddell@swin.edu.au; 3 Centre for African Conservation Ecology, Nelson Mandela University, Port Elizabeth 6034, South Africa

**Keywords:** agriculture, cluster fence, conservation fencing, dingo, feral goat, Environmental Protection and Biodiversity Conservation Act 1999, threatened species

## Abstract

**Simple Summary:**

Globally, there is a need to preserve threatened species before they disappear. The management of these species is often aided, particularly in Australia, by the addition of exclusion fences that prevent the movement of invasive or pest predators and competitors into the conservation area. Widespread use of conservation fences is limited by the availability of suitable conservation land and the relatively high costs of such fencing. Here, we discuss the potential conservation benefit of pest exclusion fences erected on agricultural land. We assess the spatial overlap of existing agricultural exclusion fences (known as “cluster fences”) with the potential habitat of listed threatened species and consider whether or not identified threats to these species are potentially alleviated within cluster fences. We find that there are several species that face threats which may be alleviated with cluster fences and propose that active recovery of threatened species on fenced agricultural land be seriously considered.

**Abstract:**

The global effort to conserve threatened species relies heavily on our ability to separate these species from the processes that threaten them, and a common tool used for this purpose is exclusion fencing. In Australia, pest animal exclusion fencing has been repeatedly used on conservation land on a small scale to successfully exclude introduced predators and competitors from threatened native fauna populations. However, in recent years, “cluster fencing” on agricultural land has re-emerged on a large scale and is used by livestock producers seeking to reduce predation losses by dingoes (*Canis familiaris*) and manage total grazing pressure from native and introduced herbivores, including red kangaroos (*Osphranter rufus*). Given that the primary threats to at-risk native fauna are also predation and overgrazing, there may be potential for cluster fencing on livestock land to achieve additional fauna conservation benefits. Understanding the amount, location and potential conservation value of cluster fenced livestock land is critical for determining how these areas might contribute to broader threatened fauna recovery goals. Drawing from publicly available databases maintained by the Australian Government, we assessed the spatial overlap of threatened species’ distributions with 105 cluster fences erected in Queensland since 2013, which cover 65,901 km^2^ of land. These cluster fenced areas represent 18 biogeographic subregions and may contain 28 extant threatened mammals, birds and reptiles including 18 vulnerable species, 7 endangered species and 3 critically endangered species. An average of nine threatened species or their habitats were identified per cluster, and over three quarters (78.6%) of these species face at least one threat that is being mitigated within clusters. The true status of threatened and pest species within clusters is largely unknown or unrecorded in most cases, but some examples of pest eradication and threatened species recovery are already emerging. Given the vast size of the cluster fenced estate, the many different biomes and species that it represents and the nature of the threats being removed within these fenced areas, we contend that agricultural cluster fencing may offer an unprecedented opportunity to advance threatened fauna conservation goals for some species at scales previously thought impossible and should be a research priority for threatened species managers.

## 1. Introduction

Globally, there is a need to manage wildlife species, both to alleviate economic pressures caused by invasive or pest species and also to mitigate anthropogenic pressures on threatened wildlife. Australia has the highest number of mammal extinctions worldwide and many other species have experienced rapid declines in abundance or distribution [1]. This decline is largely attributed to competition and predation from introduced species such as feral goats (*Capra hircus*), European red foxes (*Vulpes vulpes*) and feral cats (*Felis catus*) [2,3,4]. Many of Australia’s most at-risk species are on the brink of extinction and exist only within fenced conservation reserves or on offshore islands [5]. Exclusion fencing has become a common tool to alleviate the pressures that introduced or invasive pest animals have on native species [6]. Fences create a hard boundary around the control/management area and prevent the immigration of undesirable pest species following their removal. Fences of this style have been deployed worldwide to conserve a wide variety of species from Australian bilbies (*Macrotis lagotis*) to African elephants (*Loxodonta africana*) (e.g., [7,8]). Exclusion fencing has also been utilised globally for the benefit of agricultural industries, excluding pest species that economically affect productivity. The applications of these fences have been broad and include fencing multiple species out of large portions of countries and continents [9,10] through to fencing single species out of small exclosures [11,12,13,14].

Within Australia, exclusion fencing has been successfully employed for the conservation of small mammals, birds and reptiles [15,16,17]. The feral cat and red fox were introduced into Australia shortly after European settlement and spread quickly to occupy the majority of the country [18,19]. These two mesopredators have been repeatedly linked to extinctions and other negative effects on populations of native mammal species [2,20,21], with weights between 35 and 5500 g [22]. For this reason, the Australian Government has identified predation by cats and foxes as key threatening processes [23]. Dingo (*Canis familiaris*) predation also represents a key threatening process to many threatened fauna [24,25,26], as does competition with vertebrate herbivores including feral goats, feral pigs (*Sus scrofa*) and European rabbits (*Oryctolagus cuniculus*) [27]. Consequently, these predators and competitors of threatened fauna are actively removed and managed within almost all fenced conservation reserves in Australia [28,29], which totalled ~360 km^2^ of protected land in 2017 [30] and has since increased to 594 km^2^. Many threatened species rapidly recover when these threats are removed [5,31,32].

**Figure 1:**
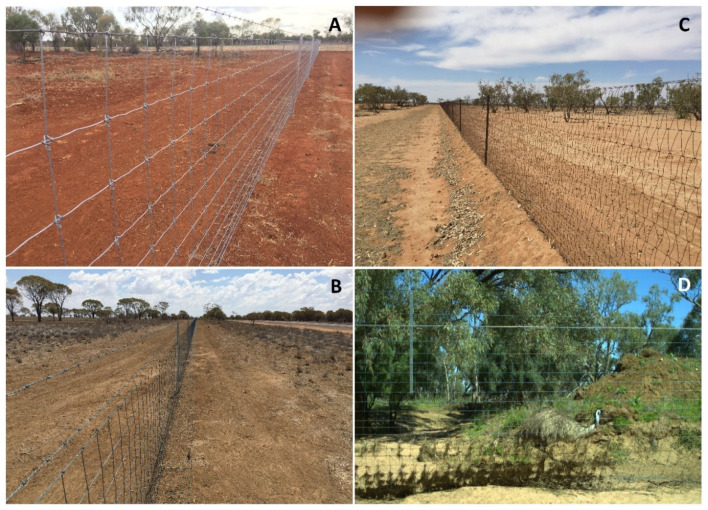
Examples of pest exclusion fence designs on agricultural lands. Examples are of netted cluster fence types in Western Queensland, showing strained aprons with one or two barbed top-wires (**A**,**B**) and those without top wires and buried aprons (**C**). A typical creek crossing is also shown (**D**), with an excluded emu.

Exclusion fences have also been used effectively in Australia for over 100 years to exclude species that cause negative economic impacts to agricultural enterprises [9]. Pest animal barrier fences were erected throughout much of Australia in the late 1800s and early 1900s [33] to slow the expansion of rabbits and exclude dingoes and emus (*Dromaius novaehollandiae*) from areas of high agricultural productivity. The fences assisted with eradicating or reducing these animals to manageable levels inside fenced areas within a few decades, but in the case of dingoes, after they had been removed, the fences eventually fell into disrepair and were replaced with standard livestock fencing of a kind that provides no impediment to dingoes and other pest animals [11]. Pest animal numbers then predictably increased over the following decades, and small groups of properties have again begun to surround themselves with pest animal exclusion fencing, colloquially known as “cluster fencing”, as they encompass a cluster of properties [34]. Such fences are netted (i.e., “hinge-joint” or “ring-lock” fencing), ~1.8 m high and typically feature a strained 30–50 cm apron extending away from the base of the fence along the ground, though sometimes this apron is buried (Figure 1). As it was historically, the goal of cluster fencing is to facilitate local eradication or suppression of agricultural pest animals inside the fences while inhibiting reinvasion from animals on the outside. It is worth noting here that feral cats are not pests to agriculture and are not controlled by cluster fencing. In Central and Central-Western Queensland, the primary target species for exclusion are dingoes and kangaroos (Macropod spp., most commonly red kangaroos *Osphranter rufus*). Dingoes and kangaroos, as well as secondary target species including feral pigs, feral goats and foxes, are considered pests to agriculture. The dingo is considered a pest species on agricultural land because of its proclivity to kill livestock, particularly sheep [11,35]. Kangaroos and feral goats are considered pest species because they contribute substantially to total grazing pressure (TGP) and land degradation [36,37,38]. The lethal control of these species is widespread within cluster fences [39] which, by 2019, now encompass ~66,000 km^2^ of protected livestock grazing land in Central-Western Queensland alone (see below).

Though their motivations and objectives are ostensibly different, conservation fencing and agricultural cluster fencing share a common enemy—vertebrate predators and competitors and the management of TGP and land degradation. Both efforts also have a long track record of achieving their objectives, having removed pest animals and conserved livestock or threatened fauna. However, while conservation fencing has demonstrated its value on small scales, there are several limitations hindering its larger-scale use, including the cost of construction and maintenance [15] and the unavailability of suitable land not already used for other purposes (see [40]). Utilising agricultural land already being pest fenced could alleviate both of these limitations (as discussed in [39]). However, no information presently exists about the potential utility of cluster fenced areas to threatened fauna conservation objectives in Australia. Understanding the location of existing cluster fences, the biomes they represent and the status of and threats to extant threatened fauna within these fences could advance the implementation of threatened fauna recovery actions on a scale previously thought impossible.

Here, we explore the potential utility of cluster fenced areas for fauna conservation by assessing the threatened species thought to be present within the recently established cluster fences in Central-Western Queensland. Our aim is to demonstrate the variety of ecosystems and threatened species that these cluster fences represent and identify the threatened species that may potentially benefit from the pest animal and land management activities occurring within these clusters.

## 2. Materials and Methods

**Figure 2:**
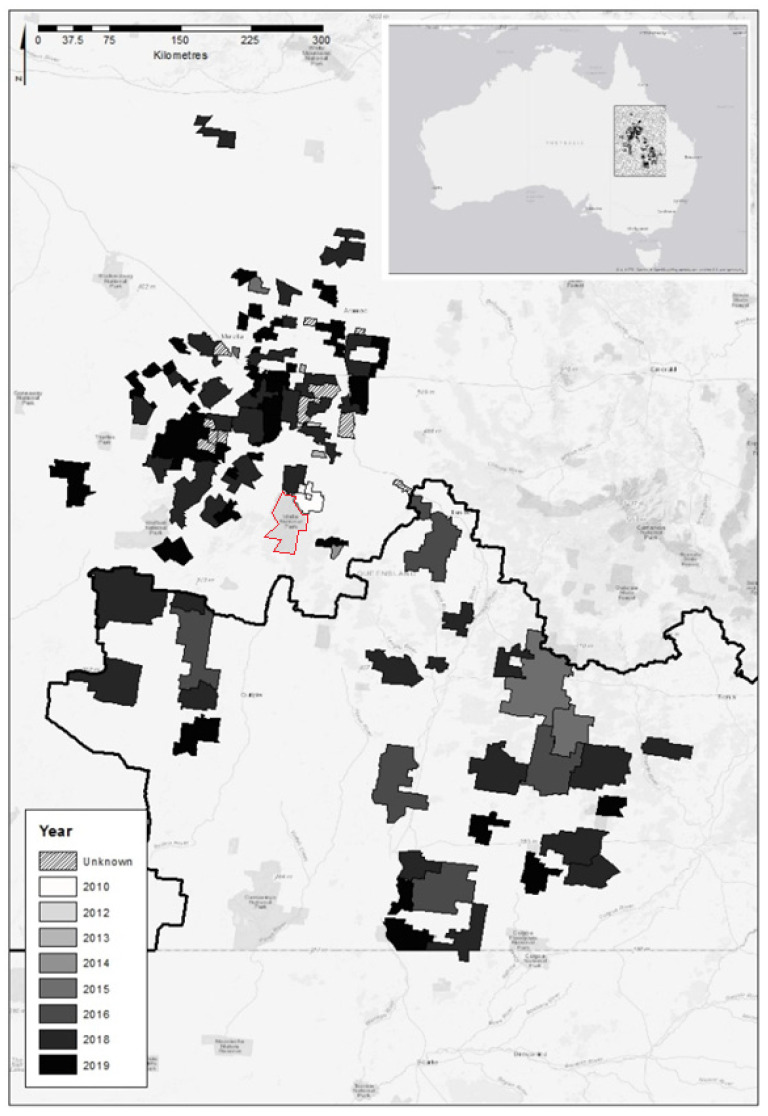
Map of pest exclusion or cluster fenced areas in Central-Western and South-Western Queensland as at December 2019. Shaded by final funding year or known year of completion. Unknown completion years of privately funded exclusion fences are indicated by hatching. Black line shows the location of the national dingo barrier fence (designed to help manage dingoes in the southern part of this area). Map generated in ArcMap v10.5.1. Red polygon shows Idalia National Park (see Discussion for details).

The names, locations, size (km^2^) and estimated completion dates of cluster fences in Western Queensland were sourced from publicly available maps provided by the government agencies that funded the construction of the cluster fences. The individually fenced clusters of properties were then geolocated using ArcMap v10.5.1 and the “Rural properties—Queensland” dataset [41] (Figure 2). If the size of a cluster was unreported, the size was projected using the “calculate geometry” tool within ArcMap based on the coordinate system used (GDA2020 MGA—Zone 55) and specified polygons (in this case, cluster fence boundaries). Next, cluster fence boundary data were used to determine which biogeographic regions and subregions were represented within the clusters using the “Biogeographic sub regions—Queensland” dataset [42]. The GPS coordinates for each cluster’s centroid were then used in the Australian Government’s Protected Matters Search Tool (PMST) interactive map (available at www.environment.gov.au) to generate a Protected Matters Report which listed extant threatened and pest fauna and their habitat, known or thought to be present within each cluster. The search radius of the PMST was limited to 10 kilometres, meaning that threatened fauna or their habitats within an area of 314.2 km^2^ around the cluster centroid were identified. From exclosure sizes, the percentage of cluster fenced land that each species might occupy was also calculated, with the assumption that the species may be present in the entirety of any cluster it was recorded in, as shown in Table 1. Given that this assumption is likely unsupported for many species, particularly for habitat specialists, more accurate distributions of some (but not all) species were able to be generated through the “Modelled potential habitat for selected threatened species—Queensland” dataset [42] (see also Table 1).

Once identified, the listed threats to each species were extracted from the SPRAT (species profile and threats) database (also available at www.environment.gov.au). We categorised and considered the relevance of whether each threat may be alleviated with clusters. These threats were dietary competition (relevant), dietary competition (irrelevant), habitat degradation (relevant), habitat degradation (irrelevant), predation by a controlled species, predation by an uncontrolled species, altered fire regimes, habitat loss, exotic weeds and human disturbance. “Irrelevant” threats were those that would not likely be mitigated within a cluster, such as degradation or competition from livestock species. “Relevant dietary competition” (i.e., competition with a controlled pest), “relevant habitat degradation” (i.e., degradation caused by a controlled pest) and “predation by a controlled species” are the threats most likely to be alleviated within cluster fences; so, we assessed which extant threatened species within cluster fences are most likely to benefit from the reduction or removal of these threats. We focused our attention on the threats being directly managed within cluster fences (e.g., predation and competition by a controlled species) even though most other threats (e.g., fire, habitat loss, weeds and human disturbance) are also being managed or mitigated indirectly.

## 3. Results

The former South-West Natural Resource Management Board (SWNRM; now known as Southern Queensland Landscapes) administered three fence funding rounds beginning after 2010. SWNRM reported that 28 clusters were fenced over the three rounds of funding, totalling 39,773 km^2^ of land located south of the dingo barrier fence (Figure 2). Most of the larger, original clusters within the SWNRM area have since been subdivided into dozens of smaller fenced areas or cells, but these have not been separately assessed here. The Remote Area Planning and Development Board (RAPAD) reported an additional 32 clusters over three funding rounds, totalling 18,461 km^2^ of land located north of the dingo barrier fence. The additional 27 clusters that sourced funding from the Longreach Wild Dog Exclusion Fence Scheme (LWDEFS) and a further 18 known, privately fenced clusters were also included in the dataset, adding another 3782 km^2^ and 3885 km^2^ to the total cluster fenced area, respectively. Thus, we assessed 105 individual clusters representing approximately 65,901 km^2^ of pest fenced agricultural land as at December 2019 (Figure 2).

**Figure 3:**
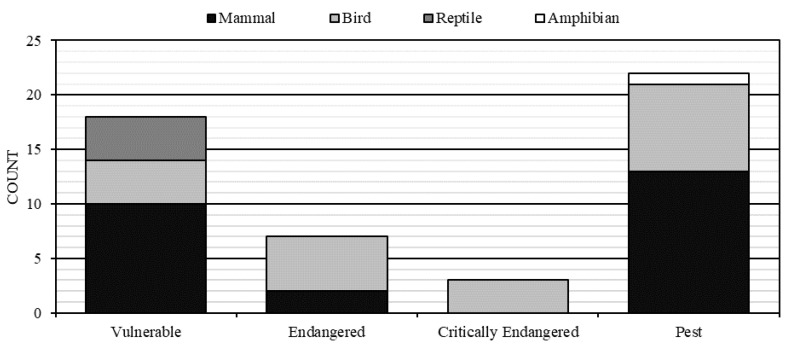
Count of fauna species identified in each class across conservation status. Bar chart presenting the count of identified species at each level categorised by Species Profile and Threats database: critically endangered, endangered, vulnerable or pest species (x axis). Columns divided by species class. Black—mammal, light grey—birds, dark grey—reptiles, white—amphibian.

The 105 clusters included parts of 18 biogeographic subregions (Figure S1), and 28 threatened species or their habitat were identified using the PMST. These included 18 vulnerable species, 7 endangered species and 3 critically endangered (CE) species (Figure 3). These three CE species—the curlew sandpiper (*Calidris ferruginea*), plains wanderer (*Pedionomus torquatus*) and eastern curlew (*Numenius madagascariensis*)—feature in 100%, 18% and 1% of PMST reports, respectively; the proportion of the cluster fenced estate that these species could occupy was 100%, 35% and 4% of cluster fenced land, respectively (Table 1). Detailed models of habitat distribution were available for 21 of the 28 threatened species (Table 1). Of note, 4031.9 km^2^ of yellow-footed rock wallaby (*Petrogale xanthopus*) habitat and 686.7 km^2^ of Julia Creek dunnart (*Smithinthopsis douglasi*) habitat is available within cluster fenced areas (Table 1, Figure S2). Additionally, 1 amphibian, 8 avian and 13 mammalian pest animals are listed as being present within clusters (Table 1, Table S1). Pest predators (or their habitat) occurring in high proportions of fenced land (based on PMST reports) were the dingo (50%), feral cat (94%), fox (96%) and the invasive herbivores feral goat (95%), feral pig (96%) and rabbit (97%).

**Figure 4:**
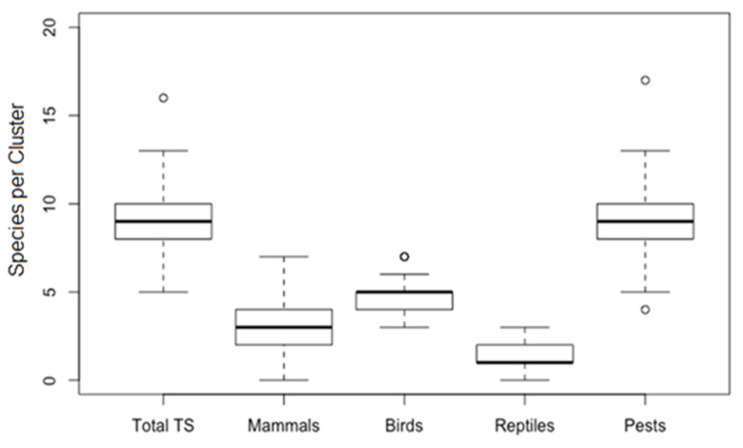
Boxplot showing count of threatened species per cluster by phylum. Included is the total threatened species (TS) per cluster and total pest species per cluster. Bold line shows median value, boxes show interquartile range. Outlier values shown as open circles. Average TS per cluster is 9.09 (SE = 0.19); mammals (mean = 2.8, SE = 0.09); birds (mean = 4.9, SE = 0.08); reptiles (mean = 1.3, SE = 0.07); pests (mean = 9.3, SE = 0.20).

**Figure 5:**
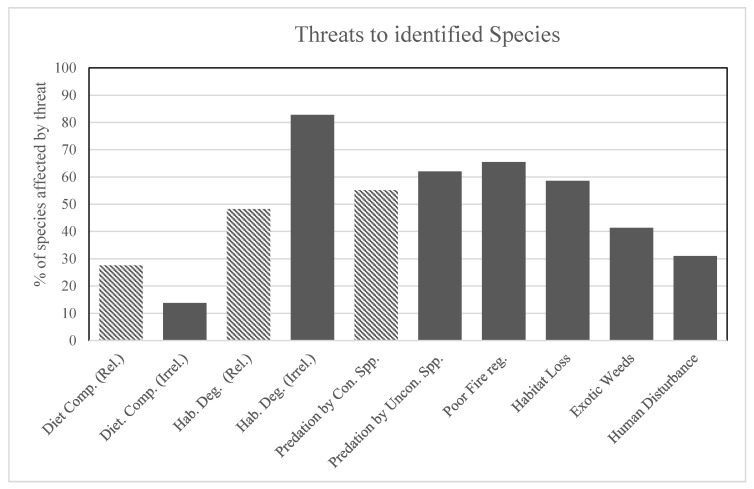
Threats to identified species. Threats recorded from individual species SPRAT file (Department of Environment and Energy). Patterned entries indicate the primary threats being actively mitigated within clusters; threats like fire, habitat loss, weeds and human disturbance are also mitigated within clusters but are not the focus of clusters.

An average of nine (SE. = 0.19) threatened species and nine (SE = 0.20) invasive species were identified per cluster (Figure 4). All documented threatened species have a variety of threatening processes listed in the SPRAT database, most commonly “irrelevant habitat degradation” (72.4%), “altered fire regimes” (65.5%) and “habitat loss” (58.6%). We found that “predation by a controlled species”, “relevant habitat degradation” and “relevant dietary competition” were recorded as threats for 55.2%, 48.2% and 27.6% of threatened species, respectively. Over three quarters (78.6%) of identified threatened species experience at least one threat that may be alleviated within clusters. Four identified species (14.3%) (night parrot *Pezoporus occidentalis*, Julia Creek dunnart, yellow-footed rock wallaby and bridled nail-tail wallaby *Onychogalea fraenata*) face all three relevant threats, a further 28.6% face two relevant threats and 35.7% face one relevant threat (Figure 5).

## 4. Discussion

There has been a rapid resurgence of pest-proof netting fencing on agricultural land across Central-Western Queensland in recent years. Since the first two cluster fences were completed in 2013 near the towns of Tambo and Morven, there are now over 105 clusters of livestock properties, totalling ~66,000 km^2^, that are enclosed by fences intended to exclude dingoes, kangaroos, feral pigs, feral goats, foxes and some other animals (Figure 2). The true status of each of these species within clusters is largely unknown or unrecorded in most cases. However, anecdotal case studies (e.g., [43]) and limited in-progress fauna monitoring data collected since 2013 (B. Allen, unpublished data) indicate that wild dogs, foxes and feral cats are either absent or at near undetectably low densities in many clusters, as are feral pigs, feral goats and rabbits. Kangaroos have also been reduced by 90–95% of their former densities in some clusters [44]. These pest removals and declines have the potential to benefit some of the 28 threatened fauna thought to be present within these cluster fences, including the eight endangered or critically endangered species (Figure 3). The predation and competition threats that are being actively alleviated within cluster fencing could potentially benefit at least 22 of the 28 (78.6%) threatened fauna known or expected to be present inside these cluster fences. Although not all land or habitats within cluster fences will be suitable for each threatened species, our results indicate that, in many cases, hundreds or even thousands of square kilometres of land within cluster fences is suitable for some species, provided that their key threats have indeed been eliminated or neutralised (Table 1). The vast size of the cluster fenced estate, the many different biomes and species it represents and the nature of the threats being removed within these fenced areas offer a remarkable opportunity to potentially advance threatened fauna conservation goals. Recovery of extant threatened fauna and/or reestablishment of locally extinct threatened fauna within cluster fences could dramatically increase global populations of these species [39].

The high cost of conservation fences remains a limiting factor in their application and extensive research has gone into developing low-cost and effective fencing designs [15,45,46,47]. Depending on the materials used and the topography of the land, conservation fences typically cost around $15,000 to $18,000 per kilometre, whereas cluster fences cost around $5000 to $8000 per kilometre, with the primary difference being the extra effort required to exclude feral cats (e.g., a taller fence with smaller gauge mesh and a floppy top). Moreover, seldom are conservation fences erected on land that generates an income capable of paying for ongoing fence maintenance, which is typically funded by governments, philanthropy and public donations. In contrast, cluster fenced agricultural land produces incomes (i.e., sales of livestock products such as wool or red meat) that can sustainably fund ongoing fence maintenance without external support. Indeed, all government-subsidised cluster fences already feature perpetual and legally-binding fence maintenance funding arrangements, and new and additional funding arrangements are also being proposed [12]. Research on choosing cost-effective locations for conservation fences also raises valid concerns about constraints on finding suitable locations that minimise outlaid economic costs and maximise species conserved [40]. A major reason for the unavailability of suitable conservation land is that it is being used for extensive livestock grazing; approximately 50% of Australia’s land mass is used for this purpose [4]. However, what if this livestock land was made suitable for conservation? Using cluster fences as pseudo-conservation reserves would be a “land sharing” initiative that has the potential to help relieve each of these issues. If current cluster fences are suitable for a given threatened species, then no additional fencing need be constructed. However, if current cluster fences require “upgrading” to be suitable for a given threatened species (e.g., to make them “cat-proof”), then conservation agencies might consider partnering with livestock producers to establish “conservation fences” for a fraction of the cost of a new conservation fence and at scales much larger than is typically possible for conservation agencies. Fence construction savings might then be used towards eradication of cats within fences, which is not a priority activity undertaken by livestock producers. The ~66,000 km^2^ of cluster fenced land identified here dwarfs the ~360 km^2^ of land contained within all the high-security fenced conservation reserves dotted across Australia [30], and these are just those cluster fences that exist in Central and Central-Western Queensland, which does not include the rapidly growing number of cluster fences in other states including New South Wales, South Australia and Western Australia. The 105 cluster fenced areas that we assessed also cover a variety of different biogeographic subregions (Figure S1), not all of which are fully represented in the national reserve estate [5]. In the effort to improve agricultural productivity, livestock producers have potentially handed conservationists a powerful tool in the fight against threatened species decline.

Given that some predators (i.e., feral cats) and competitors (i.e., livestock) may persist in some cluster fenced areas, cluster fences are probably most suitable for larger-sized threatened species and/or those that are able to tolerate low levels of predation and competition; they are unlikely to be suitable for highly threatened species that cannot tolerate any predation at all. For example, the yellow-footed rock wallaby (YFRW) is a “vulnerable” species present in 55% of PMST reports; 4031 km^2^ of cluster fenced land represents suitable habitat for them (Table 1), and they have been recently confirmed as present in several clusters (D. Smith, unpublished data). Three key threats to this species are directly eliminated or reduced within cluster fences, and four other threats are indirectly alleviated (Table 1). The conservation advice for YFRW lists predation by foxes and cats, and dietary competition and land degradation by feral herbivores and livestock, as the key threats to the species [48]. Dingoes also threaten YFRW [24] but do not appear as threats in most previous conservation advices because dingoes had been effectively eradicated and were absent from YFRW habitat for many decades at the time that this advice was generated. Dingoes are known to predate other rock wallaby species [49,50]. Control of canid predators has yielded positive results for analogous rock wallaby species [51] and fox and goat control across populations of the South Australian subspecies of YFRW (*P. x. xanthopus*) resulted in increases in abundance and distribution [52,53]. For this species, at least, recent creation of cluster fences within the present distribution of YFRW and the subsequent removal of their key threats within these fences are likely to dramatically benefit them. Using the estimates of species density calculated across 40 km^2^ of similarly suitable habitat [54], the total cluster fenced area in Queensland could potentially hold approximately 23,000 YFRW, which is more than double the current YFRW population estimate of around 10,000 mature individuals [55]. Examples like this highlight the substantial potential gains for extant threatened species if their local threats can be effectively managed within cluster fences.

Cluster fences may also be valuable locations for reintroduction of locally extinct species if historical ranges or analogous habitat for the species are identified within cluster fenced areas. Bridled nail-tail wallabies (*Onychogalea fraenata*) occur in only 2% of cluster fenced land based on PMST reports and its modelled distribution did not fall within any cluster erected by the end of 2019 (Table 1). However, the species faces all three threats being alleviated within clusters and existing clusters cover some suitable habitats for the species within its former range [56]. The species had been successfully reintroduced in the past to the unfenced Idalia National Park, which borders some of the assessed clusters (see red polygon, Figure 2 [56]), but the effort ultimately failed years later due to drought, predation and competition—the very threats being alleviated in adjacent cluster fenced areas. Given that reintroductions of this species and others have been shown to benefit from exclusion fencing in the past and fail in its absence (see [57,58,59,60]), we contend that cluster fenced areas should be seriously considered for this and other species’ reintroductions, particularly when they enclose suitable habitats for the species. The density of wallabies within the national park was reported to be 0.3/km^2^ in 1999 and increasing [56] and up to 12/km^2^ in suitable habitats [61]. Applying these densities to just the two cluster fenced properties that share a boundary with the national park (Figure 2) shows that utilisation of these two clusters for reintroductions could increase the population by 326 to 13,052 individuals. Bridled nail-tail wallabies and yellow-footed rock wallabies are just two of the many extant threatened species that we identified that could potentially benefit from cluster fences on agricultural land (Table 1).

Our study was limited by available spatial data on potential habitat and accurate estimates of species densities from healthy populations. Habitat data represent only potential habitats within a 200 km convex hull of species records that are 50 years and younger (see metadata, “Modelled potential habitat for selected threatened species” [42]) and does not cover all threatened species identified within PMST reports (Table 1). Given that species may have experienced rapid and vast declines in their range previous to the last 50 years, such as the bridled nail-tail wallaby [56], this limited our ability to accurately determine all locations where cluster fencing could be used in specific species’ conservation. In other words, our results likely underestimate the potential value of cluster fences to threatened species. Our assessment was also confined to Central and Central-Western Queensland, a unique area of broadly similar biogeography (Mulga Lands and Mitchell Grass Downs; see [62]). Despite this, the fenced areas still enclose parts of 18 different biogeographic subregions (Figure S1) and we identified 22 threatened species that may benefit from cluster fencing within these regions. Cluster fencing on agricultural land is rapidly increasing across Australia and additional cluster fences also now occur in South-East Queensland; the North-Eastern, Monaro and Western regions of New South Wales, and also in Western Australia. Cluster fencing in these areas might also benefit additional threatened species not discussed here. These limitations mean that our results merely show the potential for extant threatened species’ recovery on agricultural lands based on theoretical responses to cluster fencing and its accompanying animal management activities. On-ground research is greatly needed to determine whether or not these theoretical predictions are realistic.

Conservationists might understandably have some reservations about the utility of agricultural land for threatened species recovery. The type of fences used for cluster fencing are not exactly the same as those used for conservation, nor are the animal management activities that occur within fenced areas the same (see [63,64,65]). Livestock producers have little incentive to control some pest species, such as feral cats, which are of great concern to threatened fauna but are of negligible concern to livestock. Cluster fences may also be considered more permeable than conservation fences for some pest species, though this may not be as great a concern as might be supposed. Dingoes, foxes and cats are each known to breach even the best conservation fences at times [66]; intruding predators have decimated at-risk species in conservation zones [58] and prey naivety has been identified as a potential driver of their vulnerability. Some have proposed that exposing species to low densities of predators may assist the development of antipredator behaviours and overcome prey naivety [67,68,69]. Pest exclusion fences could therefore be utilised for this purpose as an intermediary step between high-security reserves and completely unfenced populations (see [39]). Cluster fencing may also have adverse effects on native species, such as barriers to movement and gene flow. However, many these adverse effects also occur in conservation fencing [10]. With appropriate monitoring and use of mitigation strategies (such as metapopulation management), these adverse effects can be minimised to ensure net positive outcomes.

Some conservationists might also assume that cluster fenced agricultural land does not have equivalent conservation value to conservation fenced reserve land. Though this will undoubtedly be true in some cases, there are at least three important reasons not to glibly dismiss cluster fenced land as unsuitable for conservation purposes in many or perhaps most cases. Firstly, many current national parks, reserves and conservation fenced lands were formerly degraded livestock properties sold cheaply to conservation organisations in the last few decades because their ecosystems were no longer productive enough to produce livestock. Had these lands been productive and profitable, they would likely not have been converted to non-agricultural land uses. Hence, we should not automatically assume that land currently zoned for conservation has greater ecological value than land currently zoned for agriculture when the difference between the two might only be an administrative label. Secondly, there are often large tracts of land (e.g., 25–100 km^2^) on agricultural properties that have never been grazed by livestock or have been grazed by livestock only temporarily in the distant past. Examples include the >4000 km^2^ of rugged YFRW habitat that is rarely, if ever, grazed by sheep or cattle because such rugged areas are too difficult to muster or supply water to in quantities sufficient for ruminant livestock. Therefore, even if much or most of a cluster is currently utilised by livestock, there are often many areas within clusters that have always been and are still essentially managed as “reserves”. Thirdly, even if cluster fenced land really was not as productive or not as beneficial for threatened species as a reserve, and threatened species might never reach “reserve densities” on livestock lands, such lands nonetheless represent areas where at least some threatened species may be able to re-establish populations. Achieving lower “cluster densities” in areas where they are now locally extinct, for example, is still better than having no threatened species there at all. Cluster fenced areas could play a significant role in the recovery and enduring conservation of threatened species, even if they are “not as good” as reserves in some cases.

## 5. Conclusions

Cluster fences are rapidly being erected in Queensland and across Australia, and several species that are also common threats to wildlife conservation are being either eradicated or substantially reduced across spatial scales not seen in decades. Some clusters already anecdotally report the absence or near-absence of all these species. Declines in such pest animals are yielding economic and environmental benefits to livestock producers and could yield benefits for threatened fauna conservation as well, in the following ways:

- Creation of new locations where it is possible to change the trajectory of extant threatened species populations;
- Creation of new locations where it is possible to reintroduce locally extinct species;
- Creation of new locations suitable for improving antipredator defences and overcoming prey naivety issues;
- Addition of new biomes not currently represented in the national fenced reserve system;
- Opportunities to develop private–public partnerships to share the costs of constructing high-security conservation fences (i.e., new, large conservation fences can be erected for a fraction of current costs);
- Reduction or shifting of ongoing fence maintenance costs to sources non-reliant on government, philanthropy or public donations;
- Alleviation of threatened species’ overpopulation in some of the current conservation reserves.

Cluster fenced agricultural lands may represent a stepping stone in the effort to take threatened species from high-security reserves and re-establish free-ranging and self-sustaining populations of them in unbounded areas. Though we have described a positive and optimistic opportunity for threatened fauna conservation, almost all of this remains invalidated in situ. We therefore recommend (1) on-ground monitoring be conducted to establish the presence or absence of pests and threatened fauna in priority clusters, (2) increased effort be applied to assisting the recovery of extant threatened fauna within clusters, (3) studies that monitor the net outcomes for all biota in cluster fences take place and (4) pilot studies be conducted to reintroduce threatened species expected to be resilient to the conditions found within cluster fences. Though not all threatened species will be suitable and many issues might remain unresolved, we contend that cluster fences on livestock lands should be seriously considered a key resource in the ongoing effort to conserve and recover Australia’s many unique and threatened fauna species.

## Figures and Tables

**Table 1:** List of threatened species recorded in Protected Matters Search Tool reports and their key threats. The list of threatened species identified by PMST reports to be present within clusters. Table lists their threat level status (S): critically endangered (CE), endangered (E) and vulnerable (V). (**%**) shows the percentage of clustered land the species may appear in based on assumed 100% occupancy per cluster. (km^2^) details the area of potential species distribution in km^2^ within clusters (where data exists—QSpatial). The “potentially alleviated” threats only identify those threats being directly mitigated within clusters and do not identify the additional threats being indirectly mitigated within clusters.

	Species	S	%	km^2^	Threats	Potentially Alleviated
1	Curlew Sandpiper, *Calidris ferruginea*	CE	100.0	-	4, 8, 9, 10	-
2	Plains-wanderer, *Pedionomus torguatus*	CE	35.1	481.2	4, 5, 6, 7, 8, 9	5
3	Eastern Curlew, *Numenius madagascariensis*	CE	3.66	-	4, 8, 9, 10	-
4	Star Finch, *Neochmia ruficauda*	E	44.1	-	3, 4, 5, 6, 9	3, 5
5	Black-throated finch, *Poephila cinta*	E	10.1	4.2	3, 4, 5, 6, 7, 8, 9	3, 5
6	Australian Painted Snipe, *Rostratula australis*	E	99.5	208.3	3, 4, 5, 6, 9	3, 5
7	Night Parrot, *Pezoporus occidentalis*	E	26.0	-	1, 2, 3, 4, 5, 6, 7	1, 3, 5
8	Bulloo Grey Grass-wren, *Amytornis barbatus barbatus*	E	5.16	0	3, 4, 5, 6, 8, 9	3, 5
9	Northern Quoll, *Dasyurus hallucatus*	E	4.77	0	1, 4, 5, 6, 7, 8, 9	1, 5
10	Bridled Nailtail Wallaby, *Onychogalea fraenata*	E	1.84	0	1, 3, 4, 5, 6, 7, 9	1, 3, 5
11	Squatter Pigeon, *Geophaps scripta scripta*	V	22.9	124.5	3, 4, 5, 6, 8, 9, 10	3, 5
12	Painted Honeyeater, *Grantiella picta*	V	96.3	14,710.0	2, 3, 4, 6, 8, 10	3
13	Red Goshawk, *Erythrotriorchis radiatus*	V	37.8	0	1, 2, 4, 7, 8	1
14	Masked Owl, *Tyto novaehollandiae kimberli*	V	3.66	-	2, 3, 7, 8	3
15	Greater Bilby, *Macrotis lagotis*	V	40.9	0	1, 4, 5, 6, 7	1, 5
16	Koala, *Phascolarctos cinereus*	V	69.8	193.6	5, 7, 8, 10	5
17	Julia Creek Dunnart, *Sminthopsis douglasi*	V	34.0	686.7	1, 3, 5, 6, 8, 9	1, 3, 5
18	Corben’s Long-eared Bat, *Nyctophilus corbeni*	V	41.7	193.4	4, 5, 7, 8, 10	5
19	Yellow-footed Rock-wallaby, *Petrogale xanthopus*	V	46.3	4031.9	1, 3, 5, 7, 8	1, 3, 5
20	Semon’s Leaf-nosed Bat, *Hipposideros semoni*	V	3.66	0	4, 5, 6, 7, 8, 10	5
21	Ghost Bat, *Macroderma gigas*	V	4.47	0	1, 4, 6, 7, 8, 10	1
22	Greater Glider, *Petauroides volans*	V	3.66	-	4, 6, 7, 8	-
23	Spectacled Flying Fox, *Pteropus conspicillatus*	V	3.66	0	4, 7, 10	-
24	Bare-rumped Sheathtail-bat, *Saccolaimus nudicluniatus*	V	3.66	0	4, 7	-
25	Plains Death Adder, *Acanthophis hawkei*	V	45.5	-	3, 4, 6, 7	3
26	Yakka Skink, *Egernia rugosa*	V	49.1	2202.6	4, 5, 6, 7	5
27	Ornamental Snake, *Denisonia maculata*	V	12.9	0	3, 4, 6	3
28	Adorned Delma, *Delma torquata*	V	10.9	1.3	4, 7, 9	-

Threats identified were (1) Relevant dietary competition, (2) Irrelevant dietary competition, (3) Relevant habitat degradation, (4) Irrelevant habitat degradation, (5) Predation by a controlled species, (6) Predation by an uncontrolled species, (7) Poor fire regimes, (8) Habitat loss, (9) Exotic weeds, (10) Human disturbances.

## Supplementary Materials

The following are available online at https://www.mdpi.com/2076-2615/10/9/1550/s1, Figure S1: Biogeographic subregions represented inside cluster fences in Central-Western Queensland, Australia, Figure S2: Examples of modelled suitable habitat for threatened species, overlaid with cluster fence locations. Table S1: List of pest species identified by PMST.
