# Supplementary material for: Expansion of Vertebrate Pest Exclusion Fencing and Its Potential Benefits for Threatened Fauna Recovery in Australia
Source: Animals (Basel). 2020 Sep 1;10(9):1550. doi: 10.3390/ani10091550 (PMC7552171; doi:10.3390/ani10091550)
Supplement: Supplementary file 1 [file animals-10-01550-s001.pdf]

Supplementary material

Article title: Expansion of agricultural pest exclusion fencing and its potential benefits for threatened fauna recovery in Australia

Authors: Deane Smith, Kristy Waddell and Benjamin L. Allen

Journal: Animals

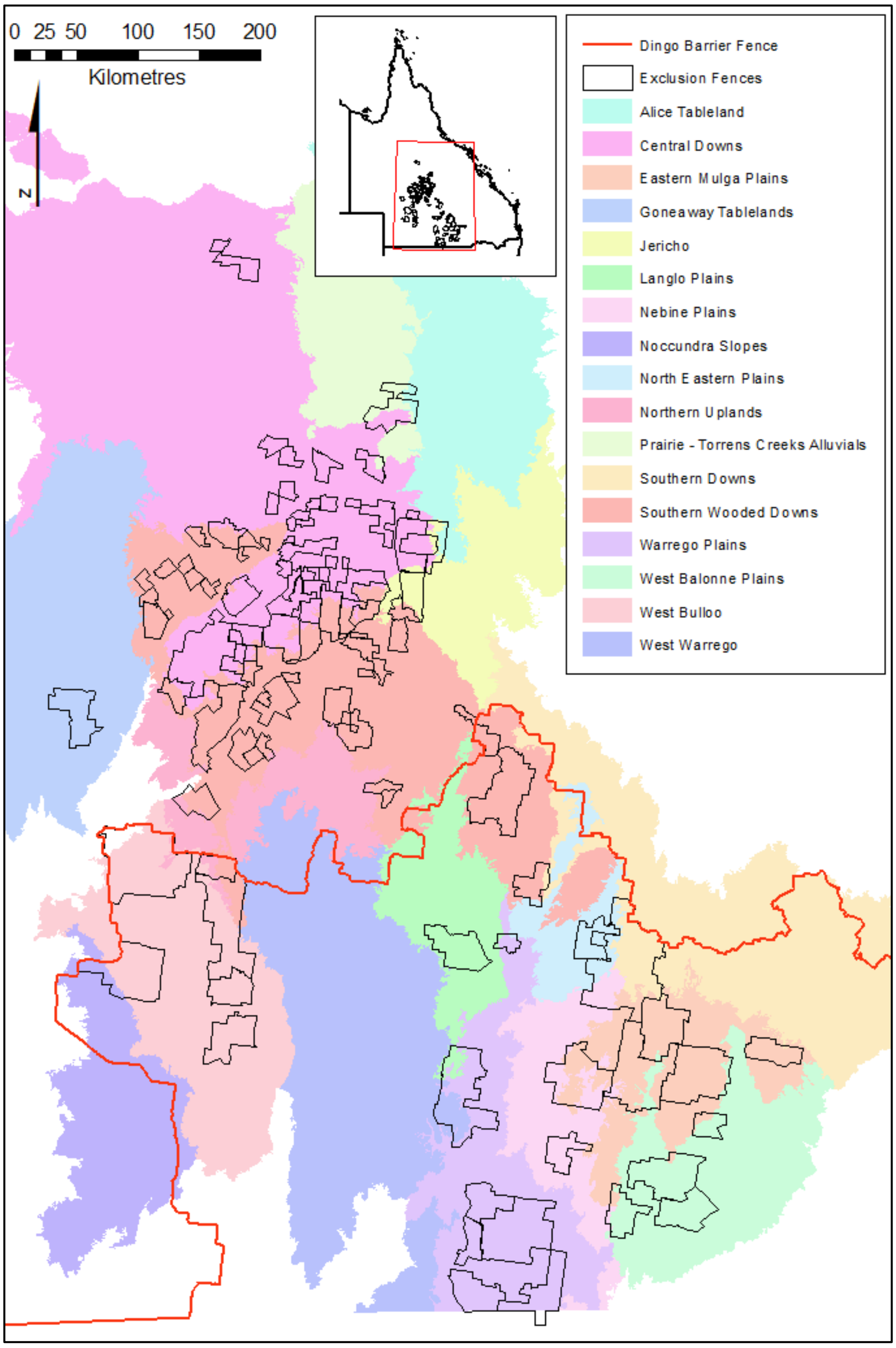

Figure S1: Biogeographic subregions represented inside cluster fences in Central-west Queensland, Australia.

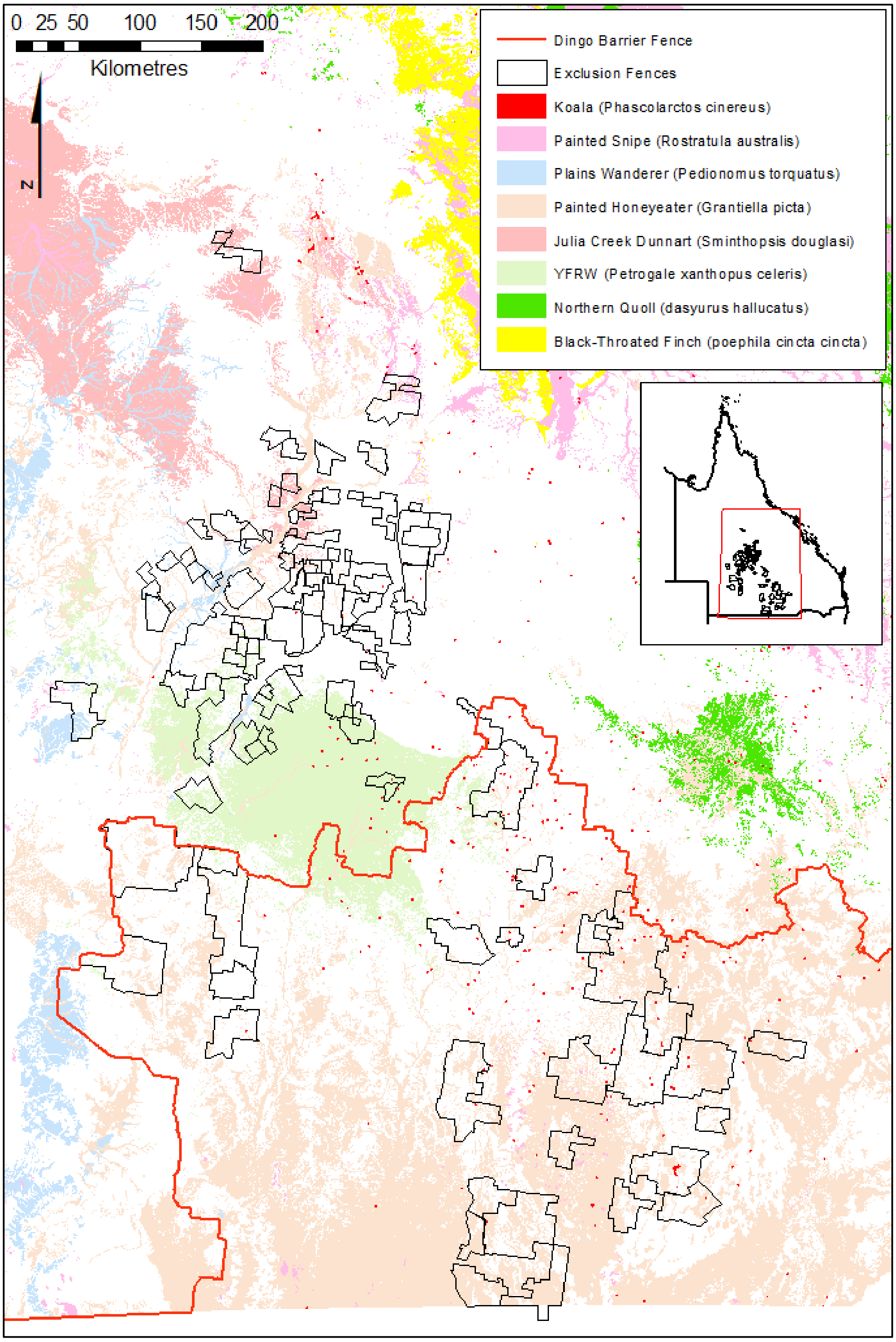

Figure S2: Examples of Modelled suitable habitat for threatened species, overlayed with cluster fence locations.

Table S1: List of pest species identified by PMST

Common name	Scientific name	Class
House Sparrow	*Passer domesticus*	Bird
Domestic Pigeon	*Columba livia*	Bird
Common Blackbird	*Turdus merula*	Bird
Common Starling	*Sturnus vulgaris*	Bird
Mallard	*Anas platyrhynchos*	Bird
Spotted turtle-dove	*Streptopelia chinensis*	Bird
Common Myna	*Acridotheres tristis*	Bird
Nutmeg Mannikin	*Lonchura punctulata*	Bird
Cane Toad	*Rhinella marina*	Amphibian
Dromedary	*Camelus dromedarius*	Mammal
Wild Dog	*Canis familiaris*	Mammal
Feral Goat	*Capra hircus*	Mammal
Feral Cat	*Felis catus*	Mammal
Red Fox	*Vulpes vulpes*	Mammal
House Mouse	*Mus musculus*	Mammal
Feral Pig	*Sus scrofa*	Mammal
Rabbit	*Oryctolagus cuniculus*	Mammal
Brown Hare	*Lepus capensis*	Mammal
Feral Cattle	*Bos taurus*	Mammal
Feral Deer	*(not specified)*	Mammal
Feral Horse	*Equus caballus*	Mammal
Black Rat	*Rattus rattus*	Mammal
